# Supplementary material for: Urban rats are the ‘fall-guy’: Resident motivations for municipal rat complaints
Source: PLoS One. 2024 Feb 8;19(2):e0296920. doi: 10.1371/journal.pone.0296920 (PMC10852225; doi:10.1371/journal.pone.0296920)
Supplement: S1 Table — The City used these categories to categorize complaints according to their contents. (DOCX) [file pone.0296920.s001.docx]

**Supporting Information for:**

**Urban rats are the ‘fall-guy’: Resident motivations for municipal rat complaints**

*Michael J. Lee^1,2^, Kaylee A. Byers^1,3^, Xiaocong Guo^1,2^, Lisa K.F. Lee^1,4^, Susan M. Cox^2,5^, Chelsea G. Himsworth^1,2,6^

^1^Canadian Wildlife Health Cooperative, Animal Health Centre, Abbotsford, BC, Canada

^2^School of Population and Public Health, University of British Columbia Canada, Vancouver, BC, Canada

^3^Pacific Institute on Pathogens, Pandemics and Society, Simon Fraser University, Burnaby, BC, Canada

^4^Department of Veterinary Pathology, Western College of Veterinary Medicine, University of Saskatchewan, Saskatoon, SK, Canada

^5^The W. Maurice Young Centre for Applied Ethics, University of British Columbia, Vancouver, BC Canada

^6^Animal Health Centre, British Columbia Ministry of Agriculture, Abbotsford, BC, Canada

*Corresponding author: Michael lee

Email: m.lee@ubc.ca

### S1 Table. Complaint categories pre-defined by the City of Vancouver. The City used these categories to categorize complaints according to their contents.

| 1. Streets – General Issues |
| --- |
| 1. Dead Animal Pickup - OLD |
| 1. Commercial Waste Container Request |
| 1. Street Cleaning & Debris Pickup Case |
| 1. Abandoned Garbage Pickup - OLD |
| 1. Cart - Garbage Case |
| 1. Animal Control General Inquiry Case |
| 1. Parks Litter Can or Cart Request |
| 1. Cart Complaint (Garbage, Green, and Recycling) Case |
| 1. Abandoned Non-Recyclables Pickup Case |
| 1. Dead Animal Pickup Case |
| 1. Street Tree Work Request – Urban Forestry Case |
| 1. General Inquiries |
| 1. Building Inspection Complaint Case |
| 1. Hoarding Concern Case – Fire |
| 1. Street Tree Work Request SR |
| 1. Missed Yard Trimmings and Food Scraps Pickup Case |
| 1. PUI Noise Complaint Case |
| 1. Street Litter Can Cleanup Case |
| 1. PUI General Inquiry Case |
| 1. General Inquiries - OLD |
| 1. Fire Safety Hazards |
| 1. Traffic Management – General Inquires Case |
| 1. Green Bin Program Feedback and Inquiry Case |
| 1. Cart – Green (Yard Trimmings and Food Scraps) Case |
| 1. Sanitation General Inquires Case |
| 1. PUI Property Use Complaint Case |
| 1. Citizen Feedback Case |
| 1. Solid Waste Management General Inquiries Case |

|  |  |  |  |  |  |  |  |  |  |  |  |  |  |  |  |  |  |  |  |  |  |  |  |  |  |  |  |  |  |  |  |  |
| --- | --- | --- | --- | --- | --- | --- | --- | --- | --- | --- | --- | --- | --- | --- | --- | --- | --- | --- | --- | --- | --- | --- | --- | --- | --- | --- | --- | --- | --- | --- | --- | --- |
|  |  |  |  |  |  |  |  |  |  |  |  |  |  |  |  |  |  |  |  |  |  |  |  |  |  |  |  |  |  |  |  |  |
|  |  |  |  |  |  |  |  |  |  |  |  |  |  |  |  |  |  |  |  |  |  |  |  |  |  |  |  |  |  |  |  |  |
|  |  |  |  |  |  |  |  |  |  |  |  |  |  |  |  |  |  |  |  |  |  |  |  |  |  |  |  |  |  |  |  |  |
|  |  |  |  |  |  |  |  |  |  |  |  |  |  |  |  |  |  |  |  |  |  |  |  |  |  |  |  |  |  |  |  |  |
