# Supplementary material for: Urban rats are the ‘fall-guy’: Resident motivations for municipal rat complaints
Source: PLoS One. 2024 Feb 8;19(2):e0296920. doi: 10.1371/journal.pone.0296920 (PMC10852225; doi:10.1371/journal.pone.0296920)
Supplement: Attachment — Submitted filename: Response to Reviewers.docx [file pone.0296920.s002.docx]

**Editor Comments:**
==============================
Authors need to explain the methodology and data more carefully for a general reader's understanding. Furthermore, the academic novelty needs to be highlighted. 
==============================

**Author Response**: Thank you for taking the time to review our manuscript. We have addressed this helpful feedback throughout the manuscript, with responses below. First, we have revised the Methods section to more clearly explain the methodology and data for general readers. We have also moved the supplementary table describing the qualitative coding process into the main text so that the methods are clear without needing to go to the supporting information. Second, we have revised and shortened the Introduction section to clarify why this study is important and to highlight its academic novelty.

When submitting your revision, we need you to address these additional requirements.

1. Please ensure that your manuscript meets PLOS ONE's style requirements, including those for file naming. The PLOS ONE style templates can be found at

<https://journals.plos.org/plosone/s/file?id=wjVg/PLOSOne_formatting_sample_main_body.pdf> and

<https://journals.plos.org/plosone/s/file?id=ba62/PLOSOne_formatting_sample_title_authors_affiliations.pdf>.

2. Thank you for stating the following in the Acknowledgments Section of your manuscript:

“This work was funded by the City of Vancouver’s 2018-2020 Street Cleaning Grant Program in support of their efforts to establish suitable rat management protocols for Vancouver.  “

We note that you have provided additional information within the Acknowledgements Section that is not currently declared in your Funding Statement. Please note that funding information should not appear in the Acknowledgments section or other areas of your manuscript. We will only publish funding information present in the Funding Statement section of the online submission form.

Please remove any funding-related text from the manuscript and let us know how you would like to update your Funding Statement. Currently, your Funding Statement reads as follows:

“This work was funded by the City of Vancouver’s 2018-2020 Street Cleaning Grant Program in support of their efforts to establish suitable rat management protocols for Vancouver. The funders had no role in study design, data collection and analysis, decision to publish, or preparation of the manuscript.”

**Author Response**: I have removed the funding information from the acknowledgements. Please keep the funding statement as is stated immediately above this response.

3. Please include captions for your Supporting Information files at the end of your manuscript, and update any in-text citations to match accordingly. Please see our Supporting Information guidelines for more information: <http://journals.plos.org/plosone/s/supporting-information>.

**Author Response**: I have included captions for the supporting information at the end of the manuscript.

4. We note that Figure 3 in your submission contain [map/satellite] images which may be copyrighted. All PLOS content is published under the Creative Commons Attribution License (CC BY 4.0), which means that the manuscript, images, and Supporting Information files will be freely available online, and any third party is permitted to access, download, copy, distribute, and use these materials in any way, even commercially, with proper attribution. For these reasons, we cannot publish previously copyrighted maps or satellite images created using proprietary data, such as Google software (Google Maps, Street View, and Earth). For more information, see our copyright guidelines: <http://journals.plos.org/plosone/s/licenses-and-copyright>.

We require you to either (1) present written permission from the copyright holder to publish these figures specifically under the CC BY 4.0 license, or (2) remove the figures from your submission:

a. You may seek permission from the original copyright holder of Figure 3 to publish the content specifically under the CC BY 4.0 license.

We recommend that you contact the original copyright holder with the Content Permission Form (<http://journals.plos.org/plosone/s/file?id=7c09/content-permission-form.pdf>) and the following text:

“I request permission for the open-access journal PLOS ONE to publish XXX under the Creative Commons Attribution License (CCAL) CC BY 4.0 (<http://creativecommons.org/licenses/by/4.0/>). Please be aware that this license allows unrestricted use and distribution, even commercially, by third parties. Please reply and provide explicit written permission to publish XXX under a CC BY license and complete the attached form.”

Please upload the completed Content Permission Form or other proof of granted permissions as an "Other" file with your submission.

In the figure caption of the copyrighted figure, please include the following text: “Reprinted from [ref] under a CC BY license, with permission from [name of publisher], original copyright [original copyright year].”

b. If you are unable to obtain permission from the original copyright holder to publish these figures under the CC BY 4.0 license or if the copyright holder’s requirements are incompatible with the CC BY 4.0 license, please either i) remove the figure or ii) supply a replacement figure that complies with the CC BY 4.0 license. Please check copyright information on all replacement figures and update the figure caption with source information. If applicable, please specify in the figure caption text when a figure is similar but not identical to the original image and is therefore for illustrative purposes only.

**Author Response**: The background map used in Figure 3 is sourced from ‘OpenStreetMaps’ ([Hyperlink](https://www.openstreetmap.org/copyright)). These maps are open data and are freely available for use under the Creative Commons Attribution-ShareAlike 2.0 license (CC BY-SA 2.0). I have included a citation for OpenStreetMaps in the revised manuscript in the figure 3 caption. This data is “free to copy, distribute, transmit and adapt … as long as you credit OpenStreetMap and its contributors.”

**Reviewer 1:**

1. Is the manuscript technically sound, and do the data support the conclusions?

The manuscript must describe a technically sound piece of scientific research with data that supports the conclusions. Experiments must have been conducted rigorously, with appropriate controls, replication, and sample sizes. The conclusions must be drawn appropriately based on the data presented.

Reviewer #1: Yes

2. Has the statistical analysis been performed appropriately and rigorously?

Reviewer #1: N/A

3. Have the authors made all data underlying the findings in their manuscript fully available?

The [PLOS Data policy](http://www.plosone.org/static/policies.action#sharing) requires authors to make all data underlying the findings described in their manuscript fully available without restriction, with rare exception (please refer to the Data Availability Statement in the manuscript PDF file). The data should be provided as part of the manuscript or its supporting information, or deposited to a public repository. For example, in addition to summary statistics, the data points behind means, medians and variance measures should be available. If there are restrictions on publicly sharing data—e.g. participant privacy or use of data from a third party—those must be specified.

Reviewer #1: Yes

4. Is the manuscript presented in an intelligible fashion and written in standard English?

PLOS ONE does not copyedit accepted manuscripts, so the language in submitted articles must be clear, correct, and unambiguous. Any typographical or grammatical errors should be corrected at revision, so please note any specific errors here.

Reviewer #1: Yes

5. Review Comments to the Author

Please use the space provided to explain your answers to the questions above. You may also include additional comments for the author, including concerns about dual publication, research ethics, or publication ethics. (Please upload your review as an attachment if it exceeds 20,000 characters)

Reviewer #1: This research has encompass the issue of rats very well. Its method are appropriate and qualitative procedures are well adopted. The researchers created a combined index of material and social deprivation using the first strategy recommended in the MSDI user guide but in results section, findings related to this index are not highlighted.
The manuscript is well written.

**Author Response:** Thank you for taking the time to review our manuscript. To address your main concern, we have further explained in the Methods section how we assessed the deprivation data. We have also more clearly highlighted the results related to the deprivation index in the Results section and in the Discussion. Overall, we found that there was some evidence that complaints were more common in areas with higher levels of deprivation. This is consistent with expectations based on the previous literature and we discuss the implications of this in section 4.3 in the Discussion.

**Reviewer 2:**

1. Is the manuscript technically sound, and do the data support the conclusions?

The manuscript must describe a technically sound piece of scientific research with data that supports the conclusions. Experiments must have been conducted rigorously, with appropriate controls, replication, and sample sizes. The conclusions must be drawn appropriately based on the data presented.

Reviewer #2: No

2. Has the statistical analysis been performed appropriately and rigorously?

Reviewer #2: No

3. Have the authors made all data underlying the findings in their manuscript fully available?

The [PLOS Data policy](http://www.plosone.org/static/policies.action#sharing) requires authors to make all data underlying the findings described in their manuscript fully available without restriction, with rare exception (please refer to the Data Availability Statement in the manuscript PDF file). The data should be provided as part of the manuscript or its supporting information, or deposited to a public repository. For example, in addition to summary statistics, the data points behind means, medians and variance measures should be available. If there are restrictions on publicly sharing data—e.g. participant privacy or use of data from a third party—those must be specified.

Reviewer #2: No

4. Is the manuscript presented in an intelligible fashion and written in standard English?

PLOS ONE does not copyedit accepted manuscripts, so the language in submitted articles must be clear, correct, and unambiguous. Any typographical or grammatical errors should be corrected at revision, so please note any specific errors here.

Reviewer #2: Yes

5. Review Comments to the Author

Please use the space provided to explain your answers to the questions above. You may also include additional comments for the author, including concerns about dual publication, research ethics, or publication ethics. (Please upload your review as an attachment if it exceeds 20,000 characters)

Reviewer #2: The authors have attempted to study an interesting issue. However, the design of the study and the nature of data don't meet the standards of sufficient academic rigor. Data analysis is limited to summary statistics.

**Author Response**: Thank you for taking the time to review our manuscript. To address your concerns about the nature of these data, the design of the study, and the use of summary statistics we have further clarified in the manuscript that this is a qualitative study in which the primary objective of the analysis was to describe the content of these textual complaints made to the City about rats. We use a method called ‘qualitative description’ which we support with references in the Methods section. This method focuses on summarizing the breadth of content in these complaints to understand why different people in the City of Vancouver cared enough about rats to complain about them. As a result, we only provide summary statistics so that readers can get a sense of the breadth of the different kinds of complaints. We do not perform further statistical analysis because these complaints were not collected from a representative sample of the general population and thus statistical conclusions could lead to erroneous inferences about the distribution of these rat problems in the wider population.
